# Cluster analysis successfully identifies clinically meaningful knee valgus moment patterns: frequency of early peaks reflects sex-specific ACL injury incidence

**DOI:** 10.1186/s40634-019-0205-5

**Published:** 2019-08-09

**Authors:** Haraldur B. Sigurðsson, Kristín Briem

**Affiliations:** Research Centre for Movement Sciences, University of Iceland, Reykjavík, Iceland

**Keywords:** ACL, Biomechanics, Cluster analysis, Data mining, Injury risk

## Abstract

**Background:**

Biomechanical studies of ACL injury risk factors frequently analyze only a fraction of the relevant data, and typically not in accordance with the injury mechanism. Extracting a peak value within a time series of relevance to ACL injuries is challenging due to differences in the relative timing and size of the peak value of interest.

**Aims/hypotheses:**

The aim was to cluster analyze the knee valgus moment time series curve shape in the early stance phase. We hypothesized that 1a) There would be few discrete curve shapes, 1b) there would be a shape reflecting an early peak of the knee valgus moment, 2a) youth athletes of both sexes would show similar frequencies of early peaks, 2b) adolescent girls would have greater early peak frequencies.

**Methods:**

*N* = 213 (39% boys) youth soccer and team handball athletes (phase 1) and *N* = 35 (45% boys) with 5 year follow-up data (phase 2) were recorded performing a change of direction task with 3D motion analysis and a force plate. The time series of the first 30% of stance phase were cluster analyzed based on Euclidean distances in two steps; shape-based main clusters with a transformed time series, and magnitude based sub-clusters with body weight normalized time series. Group differences (sex, phase) in curve shape frequencies, and shape-magnitude frequencies were tested with chi-squared tests.

**Results:**

Six discrete shape-clusters and 14 magnitude based sub-clusters were formed. Phase 1 boys had greater frequency of early peaks than phase 1 girls (38% vs 25% respectively, *P* <  0.001 for full test). Phase 2 girls had greater frequency of early peaks than phase 2 boys (42% vs 21% respectively, *P* <  0.001 for full test).

**Conclusions:**

Cluster analysis can reveal different patterns of curve shapes in biomechanical data, which likely reflect different movement strategies. The early peak shape is relatable to the ACL injury mechanism as the timing of its peak moment is consistent with the timing of injury. Greater frequency of early peaks demonstrated by Phase 2 girls is consistent with their higher risk of ACL injury in sports.

## Background

Anterior cruciate ligament (ACL) injuries result in considerable societal burden (Kiadaliri et al., 2016), explaining extensive and ongoing research efforts to prevent them. Cadaver studies have demonstrated that the ACL can be loaded through a knee valgus moment (VM) (Markolf et al., 1990), and that the VM is an important contributor to the multi-planar loads that produce clinically meaningful injury patterns (Bates et al., 2018). A landmark study by Hewett et al. (Hewett et al., 2005) revealed that the knee valgus moment was a risk factor for ACL injury, but had important limitations. The total number of injured players was low (*N* = 9), leading to a high chance of false discoveries (Christley, 2010; Colquhoun, 2014). Furthermore, the study used a bilateral drop-jump, a movement which typically does not result in athletic ACL injuries (Montgomery et al., 2018; Walden et al., 2015).

Recent studies using similar methodology (Krosshaug et al., 2016; Leppanen et al., 2017) have not replicated the results of the Hewett study (Hewett et al., 2005) and the observation has been made that biomechanical risk factor studies seldom account for the ACL injury mechanisms in their analyses (Dai et al., 2014) which may explain their inconsistent results. While ACL injuries occur shortly after contact with the ground (Koga et al., 2010; Krosshaug et al., 2007), prospective studies have extracted peak values over the complete weight acceptance phase (Hewett et al., 2005; Krosshaug et al., 2016; Leppanen et al., 2017). The timing of global peaks occur during mid- to late weight acceptance phase, which is inconsistent with that of ACL injury (Sigurethsson et al., 2018). A key difficulty in extracting the peak value of the knee VM during the critical early contact phase is the variability in the waveform of the calculated VM signal, which doesn’t always have a discrete peak in the early phase (Sigurethsson et al., 2018).

Augmenting traditional biomechanical approaches with machine learning tools, such as cluster analysis (Halilaj et al., 2018) has been suggested as a means for opening new avenues of research. Identifying a waveform consistent with the mechanism of ACL injury is a classification problem that may be solved with cluster analysis. To date, no method has been published that clusters joint moment waveforms into different shapes.

The primary aim of this study was to test the feasibility of using cluster analysis to identify different shapes of VM waveforms in the early weight acceptance phase of a change of direction task, a movement during which ACL injuries occur (Walden et al., 2015). Our hypotheses were; 1a) the waveforms may be classified into a small number of categories, 1b) at least one of the resulting clusters will have an early peak consistent with the timing of ACL injury (Krosshaug et al., 2007).

A secondary aim was to compare the frequency of the early peak waveform between the sexes before and after puberty. Our hypotheses were that; 2a) before adolescence, athletes will show an identical frequency of early peaks, 2b) after adolescence girls will have greater frequency of early peaks, consistent with the 2-3x greater risk of sports related ACL injuries reported in the literature (Montalvo et al., 2018; Nicholls et al., 2018).

## Methods

### Design and setting

Prospective cohort laboratory study.

### Subjects

Athletes were 9–12 years old at baseline (phase 1) and recruited from local soccer and team handball clubs. This age range has been shown to have identical ACL injury rates (Nicholls et al., 2018) in the country where the study is performed. At the follow up data collection (phase 2), these same athletes (some of whom have changed, or departed from, sports) were aged 14–17 years old for a mean time from baseline to follow up of 5 years. Athletes’ characteristics for phase 1 (*N* = 213, 39% boys) and phase 2 (*N* = 35, 45% boys) are summarized in Table 1.

**Table 1:** Descriptive Statistics

		Boys		Girls		P
		Mean	SD	Mean	SD	
Phase 1	Age	10.6	0.70	10.8	1.06	0.148
	Height	149.0	7.87	149.9	8.20	0.721
	Weight	40.2	8.10	41.8	9.38	0.402
	No. of Trials	1512		2502		
Phase 2	Age	15.8	0.81	16.0	0.77	0.500
	Height	180.7	9.11	167.4	3.99	< 0.001
	Weight	74.9	16.54	64.0	10.27	0.054
	No. of Trials	364		419		

No. of Trials are the number of trials collected that entered the cluster analysis process

### Data collection

Data collection methods have been previously described by Briem et al. (Briem et al., 2017). In short, height and weight were measured before a short warm-up on a stationary bike. Strength testing of hip muscles in abduction and external rotation was performed.

After strength testing, 46 reflective markers were placed on the subject, 4 on each foot, one per malleolus, a 4 marker cluster on each shank, one per femoral condyle, a 4 marker cluster on each thigh, a 3 marker cluster on the sacrum, one on each greater trochanter of the femur and on the highest point of each iliac crest, on bilateral anterior superior iliac spines, on the thorax (approximately t10-t12), on the c7, on the sternum, and on the lateral aspects of each scapular acromion.

A static trial was recorded, and anatomical markers were removed (trochanteric, malleolar, condylar, and iliac crests) before the dynamic movement trials. Subjects performed 5 repetitions of a change of direction task on each leg, and 5 repetitions of a bilateral drop-jump from a 23 cm (youth) or 30 cm (adolescents) box. Movement tasks were repeated after a 5 min skateboard exercise protocol and all conditions were pooled for this analysis. The order of movement trials was randomized with an online randomizer in phase 2 (Random.org, 2016), and a coinflip in phase 1.

### Data processing and statistical analysis

An 8 segment, 48 degree of freedom, musculoskeletal model was constructed in Visual3D (C-Motion) consisting of feet, shanks, and thighs of both lower extremities, in addition to a pelvis and a trunk. Ankle joint centers were defined as midway between malleolar markers, knee joint centers as midway between femoral condyle markers, hip joint centers as 25% of the distance between trochanteric markers, and the pelvis-trunk joint as midway between the iliac crest markers. Visual3D default settings were used for all segment inertial parameters.

Calculations of kinematics were performed using the 6 degree-of-freedom method and inverse kinetics were calculated for joint moments. Joint moments were normalized by subject body weight, since the tensile strength of the ACL ligament also scales with body weight (Chandrashekar et al., 2006). Time series data of the stance phase of a change of direction task was exported from Visual3D (C-Motion) and imported into R (Team, 2018) for analysis. Video analysis of ACL injuries have revealed that ACL injuries occur in the initial 50 ms after contact with the ground (Krosshaug et al., 2007). However, these descriptions of ACL injuries most often involve high level athletes (Koga et al., 2018) due to the availability of match video recordings. With that in mind, we observed that the fastest athletes in our cohort who displayed an early peak knee VM did so close to the 50 ms mark, which was generally within the first 25% of the stance phase. In order to ensure that slopes on either side of the peak waveform would be captured, data from the first 30% of stance were selected for the cluster analysis.

### Cluster analysis

**Fig. 1:**
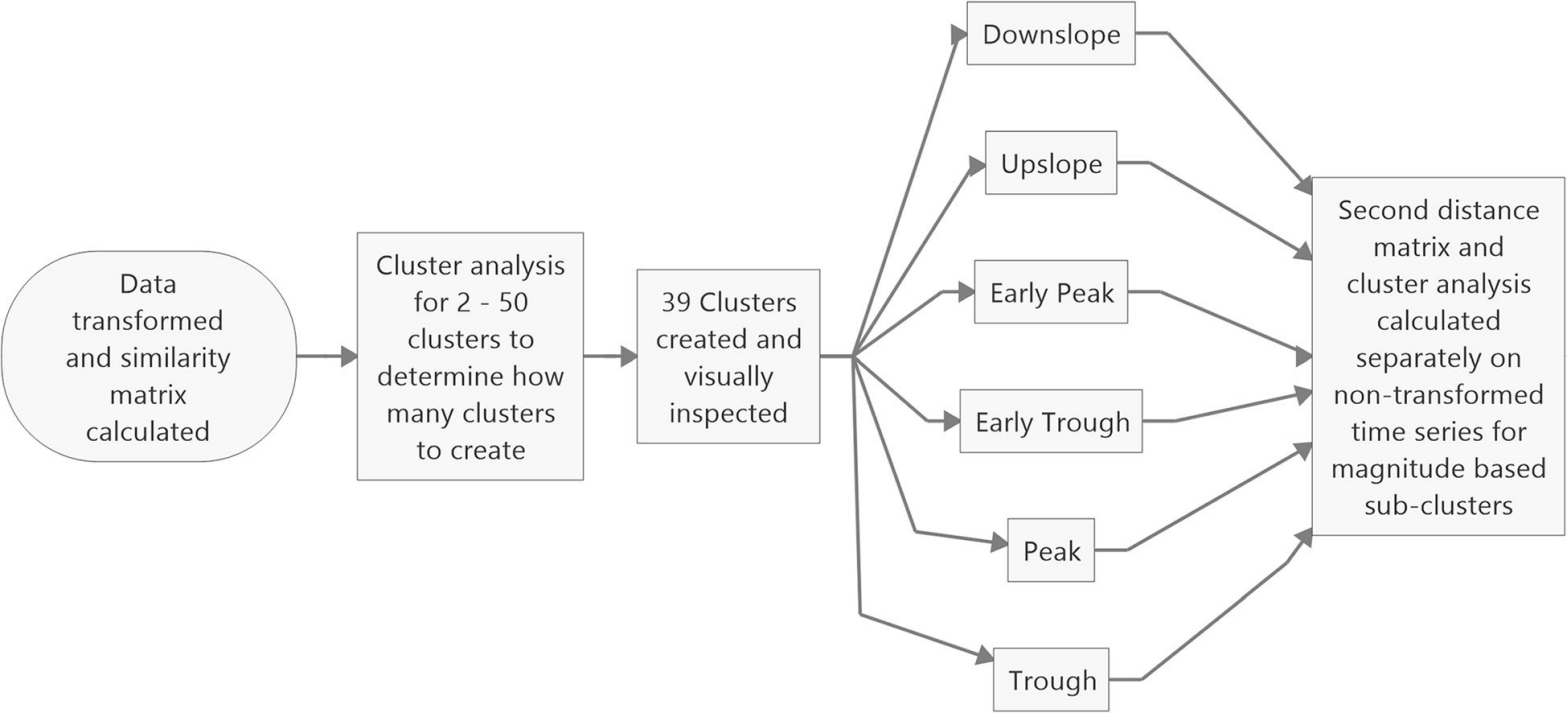
Overview of the cluster analysis process

Cluster analysis is a mathematical method which seeks to form groups of discrete data points such that they are more similar to other members within the cluster than they are to those outside the cluster. How well a data set has been clustered can be calculated as the C-Index (Hubert & Levin, 1976), which is the ratio of distances within clusters divided by distances outside a cluster. A requirement for cluster analysis is that the similarity or dissimilarity is calculated between each pair of observations. For the cluster analysis technique presented here (Fig. 1), each recorded trial entered the process separately (at most 20 trials for each athlete and phase) and the dissimilarity metric was calculated as the Euclidean distance (Montero & Vilar, 2014) between the waveforms. The method requires that each time series contains equally many data-points, and thus each time series was first interpolated to lengths equal to the longest series + 2 frames.

A transformation was then performed by calculating the lagged differences of the series and taking its sign. Thus, if a VM data point was higher than that found in the prior frame it was given the value 1, whereas if the data point was lower than that in the prior frame, a value of − 1 was given. Each time series was therefore reduced to its directional changes (increasing or decreasing), representing its waveform. The Euclidean distances between the transformed waveforms were calculated (Montero & Vilar, 2014) and clusters formed using the Ward.D2 (Charrad et al., 2014; Murtagh & Legendre, 2014) method which produces compact spherical clusters.

To decide on a number of clusters to produce, the C-Index (Hubert & Levin, 1976) was calculated for total cluster numbers from 2 to 50 clusters. As there was no distinct elbow in the C-Index plot, a number of clusters was selected based on a C-Index cut-off value of 0.05. The resulting clusters were visually examined and assigned to groups based on similarities in their appearance. Individual curves within a cluster were examined when the aggregated cluster appearance was unclear.

In order to differentiate between different magnitudes of similar shapes of knee VM data, a second cluster analysis step was performed. All curves within each shape were interpolated and divided by bodyweight in kg. The Euclidean distances between them were calculated and using the Ward.D2 method (Murtagh & Legendre, 2014), 2–4 sub-clusters based on force magnitude were formed. The lowest C-Index value out of the result was selected. Each of the resulting sub-clusters were then examined and classified as either a small, medium, or a large magnitude.

### Statistical analysis

No specific cut-offs have been commonly accepted to determine the quality of clusters formed with cluster analysis. Instead, the cluster analysis process was visually inspected to confirm that the intended goal of discrete shapes in the VM waveform was reached. For the secondary aims of determining sex- and age-dependent differences in the frequency of the early peak VM shape, a chi-square test was performed on the frequency distribution of the clusters by sex and maturity where each individual trial was the unit of study. Significance level was set at 0.05.

## Results

After screening for errors in performing the side-step maneuver as well as removing trials with large artifacts, 4903 attempts out of the 5080 collected were available for analysis.

### Clustering process

**Fig. 2:**
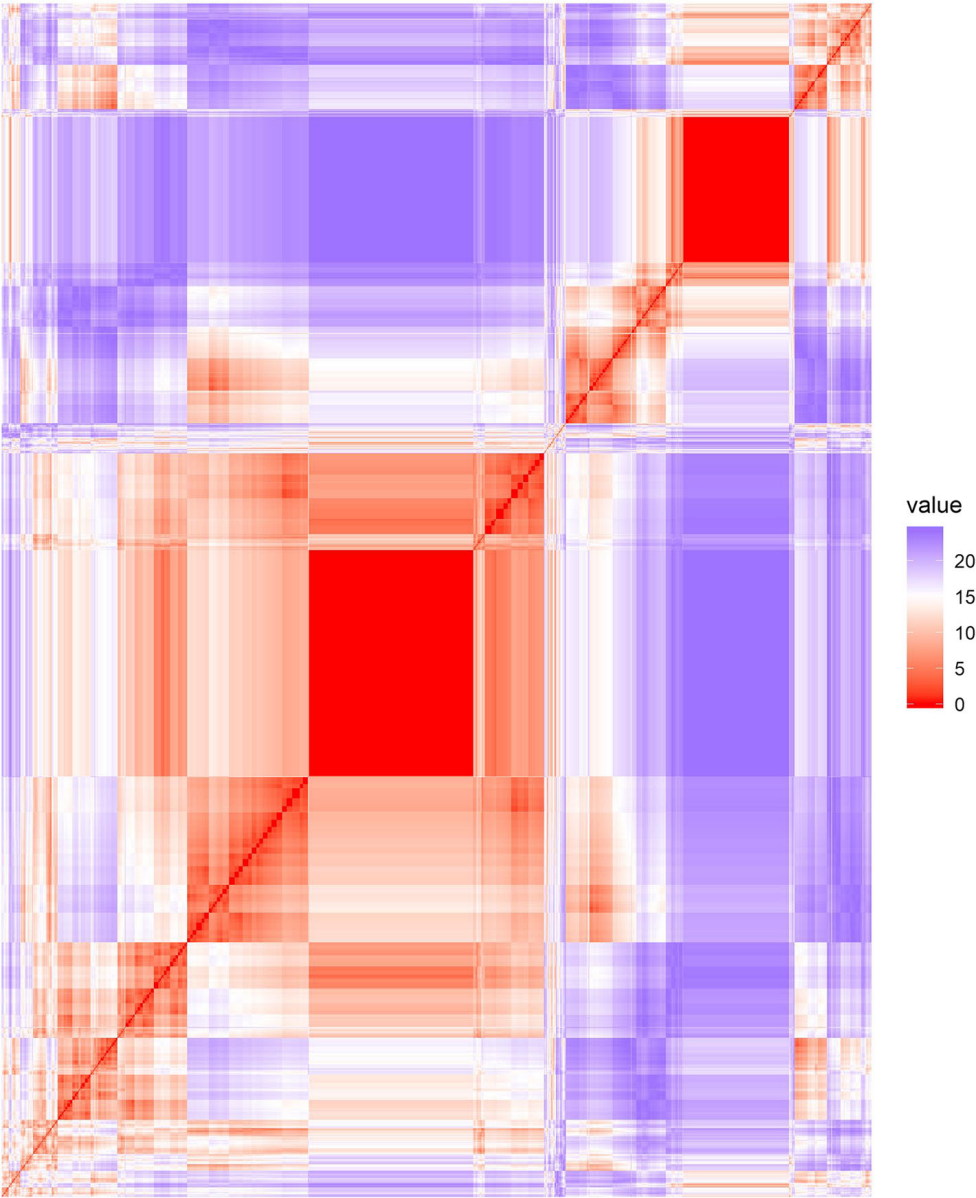
Heat map of the Euclidean distances of the time series after reduction to the signs of the differenced curve. Two large solid red boxes are present, indicating a number of identical time series (distance = 0)

**Fig. 3:**
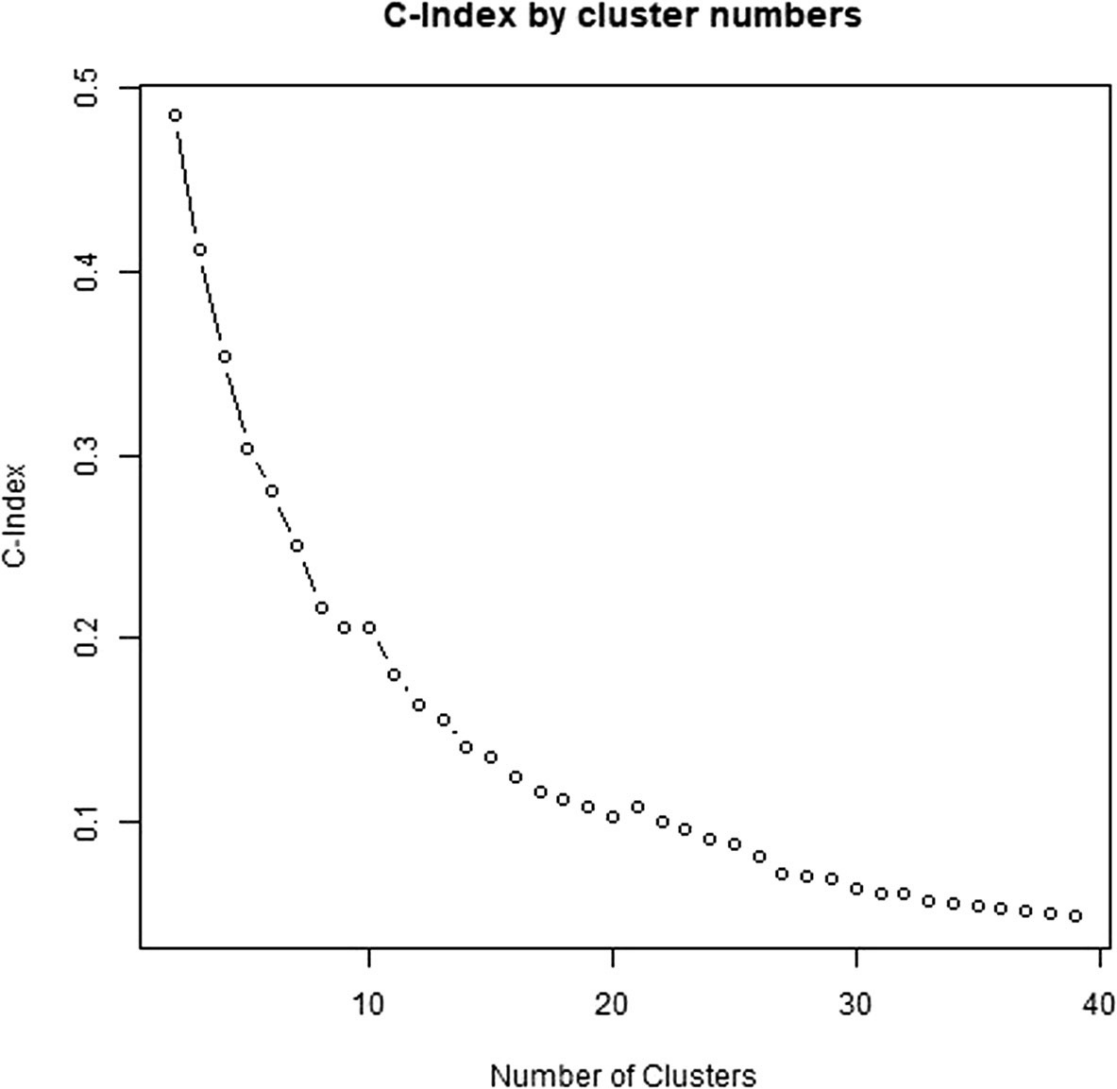
C-Index plot of initial cluster analysis step

**Fig. 4:**
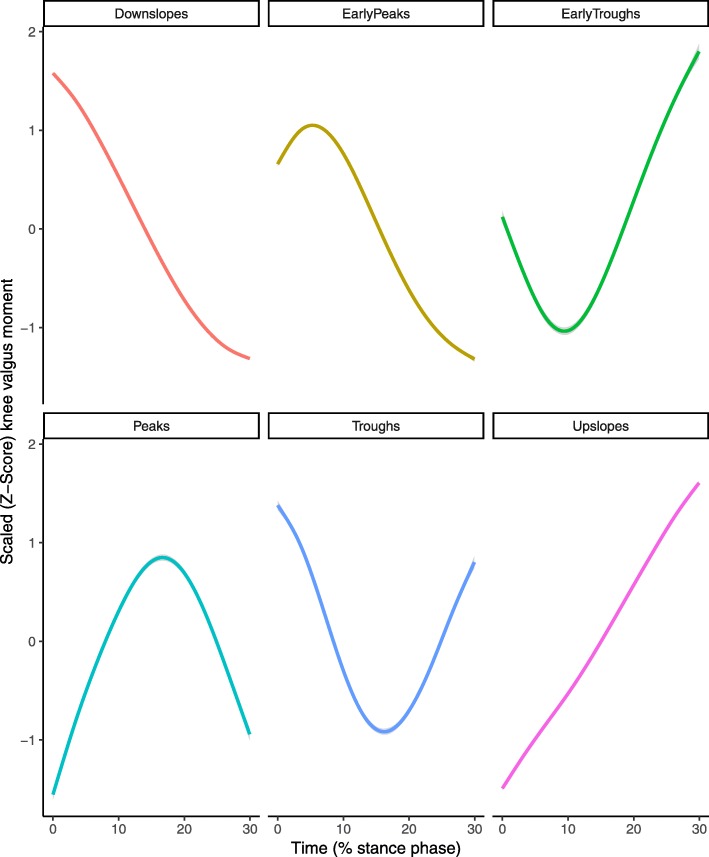
Smoothed aggregate time series’ of the six basic shapes of the scaled knee valgus moment curves generated in the initial shape-based cluster analysis. Each time series is individually scaled. The gray shaded area denotes the 95% confidence interval of the smoothing process

**Fig. 5:**
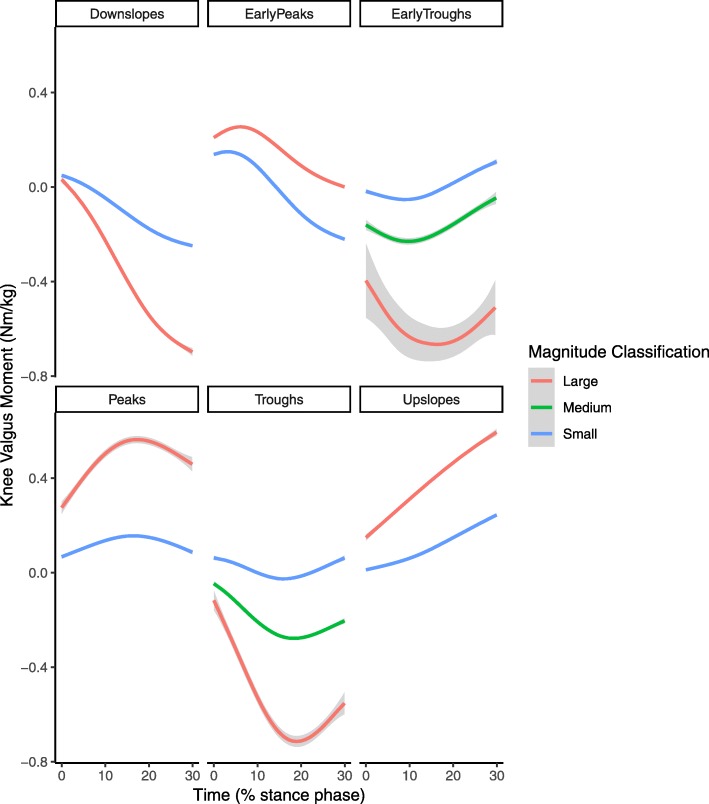
Smoothed aggregates of the time series of the first 30% of the stance phase of all clusters generated with the two step cluster analysis. The gray shaded area denotes the 95% confidence interval from the smoothing process

**Fig. 6:**
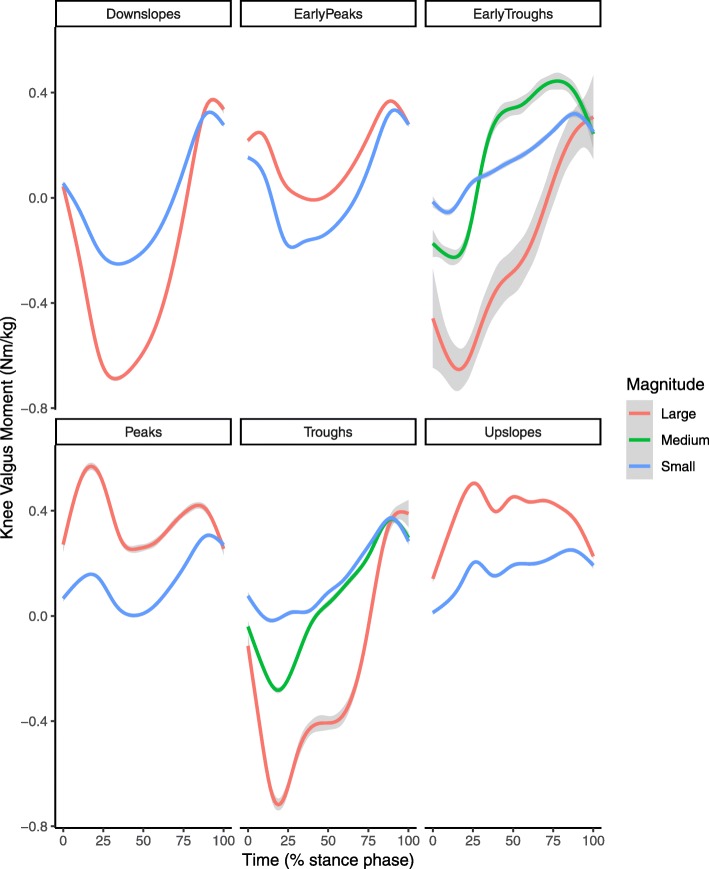
Smoothed aggregates of the time series of the whole stance phase of all clusters generated with the two step cluster analysis. The gray area denotes the 95% confidence interval from the smoothing process

After reducing each time series to the signs of a lagged difference, a total of 1025 unique shapes were present with a median of 1 trials per shape but with two large groups of identical shapes (Fig. 2). A total of 39 clusters were formed in the initial cluster analysis step. No elbow was observed in the C-Index plot and the C-Index for 39 clusters was 0.049 (Fig. 3). From those 39 clusters, 6 distinct shapes were identified (Fig. 4); early peaks, peaks, upslopes, downslopes, early troughs, and troughs. From the six basic shapes, a total of 14 magnitude based sub-clusters were formed (Figs. 5 & 6).

### Chi-squared test

The chi-squared test for the six basic shapes revealed that in phase 1, boys had a greater than expected frequency of early peaks, while girls had a lower than expected frequency (chi-square contributions of 26.4 and 20.8, respectively). In phase 2, boys had a lower than expected frequency while girls had a greater than expected frequency of early peak shapes (chi-square contributions of 10.2 and 18.9, respectively). The total Chi-Square value of the test was 400.1 with *P* < 0.001. The frequencies, expected frequencies and chi-square contributions for shapes are reported in Table 2.

**Table 2:** The observed and expected frequencies of the six shape-based clusters representing the knee valgus moment

		Observed (Expected)		Chi-square contribution	
		Boys	Girls	Boys	Girls
Phase 1	Downslopes	276 (420)	698 (695)	49.46	0.01
	Early Peaks	575 (464)	642 (768)	26.40	20.76
	Early Troughs	42 (41)	80 (67)	0.04	2.40
	Peaks	154 (149)	272 (247)	0.14	2.48
	Troughs	160 (164)	278 (272)	0.11	0.14
	Upslopes	305 (273)	532 (452)	3.68	14.08
Phase 2	Downslopes	207 (101)	152 (116)	110.77	10.87
	Early Peaks	78 (112)	178 (129)	10.20	18.92
	Early Troughs	4 (10)	3 (11)	3.42	6.07
	Peaks	10 (36)	38 (41)	18.75	0.28
	Troughs	55 (40)	28 (46)	6.05	6.74
	Upslopes	10 (66)	20 (76)	47.31	41.01

Chi-square contribution is the individual cell contribution to Chi-square value from the chi square test. *P*-value for the Chi-Square test < 0.001

**Fig. 7:**
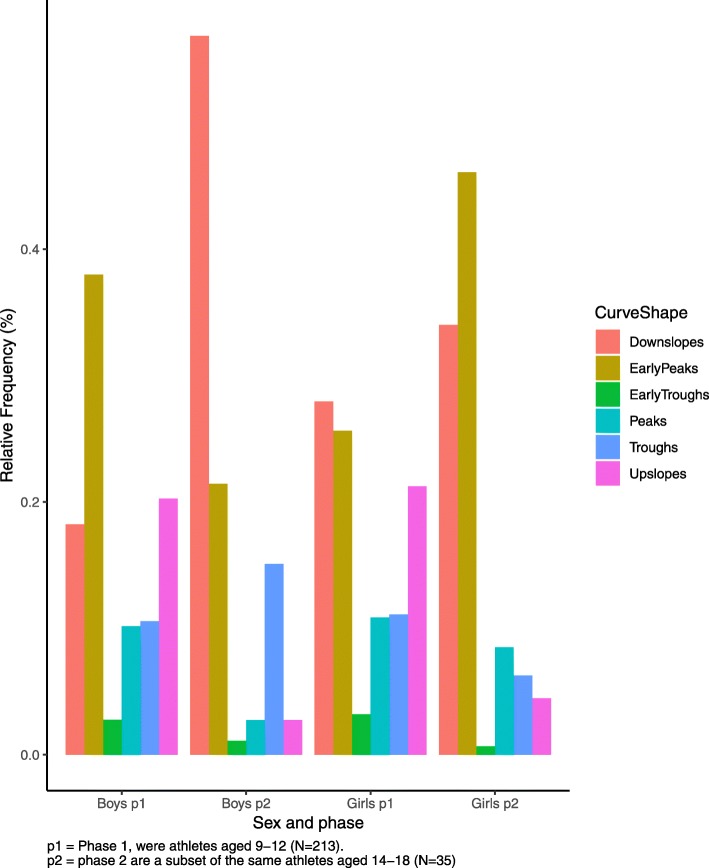
Relative frequencies of each shape according to sex and phase

The relative frequency of the early peak shape overall was 32% in phase 1 and 32% in phase 2. The relative frequencies of the sexes were such that in phase 1 boys showed an early peak in 38% of trials while girls showed an early peak in 25% of trials. In phase 2 boys showed an early peak frequency of 21% (decreased from phase 1) while girls showed an early peak frequency of 42% (increased from phase 1). The relative frequency of each shape by sex and phase are shown in Fig. 7.

During further analysis of shape and magnitude based clusters, expected frequencies for early troughs and some groups of troughs were below 5 indicating that the assumptions of the Chi-square test are violated. A Monte-Carlo simulation procedure was used as a significance test (Adery, 1968) instead. Analyses focusing on the knee VM demonstrated that phase 2 boys had fewer than expected large early peaks, while phase 2 girls had the expected frequency (chi-square contributions of 17.1 and 1.4, respectively). For small early peaks, phase 2 boys had the expected frequency while phase 2 girls had greater than expected (chi-square contributions of 0.1 and 51, respectively). The total Chi-Square value of the test was 745 with *P* < 0.001. The observed and expected frequencies with chi-square contributions for shapes and magnitudes are reported in Table 3.

**Table 3:** Observed and expected frequencies for shape based clusters and magnitude based sub-clusters

		Observed (Expected) Frequencies				Chi-Square contributions			
		Boys		Girls		Boys		Girls	
Curve shape	Magnitude classification	Phase 1	Phase 2	Phase 1	Phase 2	Phase 1	Phase 2	Phase 1	Phase 2
Early Peak	Small	291 (261)	58 (60)	335 (422)	130 (70)	3.4	0.1	18.0	51.0
	Large	284 (212)	20 (49)	307 (342)	48 (57)	24.8	17.1	3.5	1.4
Peak	Small	101 (112)	10 (26)	206 (182)	33 (30)	1.1	9.8	3.3	0.3
	Large	53 (40)	0 (9)	66 (64)	5 (11)	4.4	9.2	0.0	3.0
Downslopes	Small	198 (268)	83 (62)	478 (434)	77 (72)	18.4	7.1	4.6	0.3
	Large	78 (160)	124 (37)	220 (258)	75 (43)	41.7	205.8	5.5	24.1
Upslopes	Small	166 (167)	7 (39)	328 (269)	18 (45)	0.0	25.8	12.9	16.0
	Large	139 (112)	3 (26)	204 (180)	2 (30)	6.7	20.2	3.1	26.1
Troughs	Small	114 (110)	14 (25)	199 (177)	15 (29)	0.2	5.1	2.6	7.1
	Medium	43 (47)	24 (11)	70 (76)	9 (13)	0.3	16.0	0.4	1.0
	Large	3 (11)	17 (2)	9 (17)	4 (3)	5.4	86.5	3.8	0.5
Early Troughs	Small	31 (33)	3 (8)	67 (54)	3 (9)	0.2	2.9	3.2	4.0
	Medium	9 (7)	1 (2)	12 (11)	0 (2)	0.5	0.2	0.0	1.9
	Large	2 (1)	0 (0)	1 (2)	0 (0)	1.1	0.2	0.2	0.3

The Chi-Square value is 745 and the *P* value of a Monte-Carlo simulation significance test is < 0.001

## Discussion

The main results of this study are in line with hypothesis 1a, i.e. that the two-step clustering process reported can differentiate between six different curve shapes of the knee VM during the early stance phase, and 2–3 different magnitudes within each shape. Moreover, one of the shapes identified was the early peak, consistent with hypothesis 1b. In phase 1 boys had a greater relative frequency of early peaks, in contrast to hypothesis 2a. However, consistent with hypothesis 2b, girls in phase 2 did have a greater relative frequency of early peaks with a ratio of 2:1, consistent with the reported 2-3x higher incidence of ACL injuries for adult females (Montalvo et al., 2018; Walden et al., 2011).

The Van Mechelen model of injury prevention is an established framework to guide preventative research (van Mechelen et al., 1992). The model emphasizes the need to first establish the aetiology and mechanisms of injury before implementing interventions. Extensive research has been conducted on the mechanism of injury, including cadaver models of ACL injuries (Bates et al., 2018), but very little work has been done to discover how components of injury mechanisms are manifested in non-injury movements. This dearth of cross-sectional research has resulted in prospective studies that are largely exploratory (Hewett, 2019). The relatively low incidence of ACL injuries (Montalvo et al., 2018) means that the ACL-injured cohort in prospective risk factor studies is likely to be small, with a resulting elevated risk of both false positive and false negative results (Christley, 2010; Colquhoun, 2014). Rather than exploration, risk factors tested in prospective studies should be grounded by theory to produce more robust findings.

That is the context in which this study is placed. We present cluster analysis of knee VM waveforms as a novel method to identify the existence of an important component of the ACL injury mechanism, the early peak VM (Bates et al., 2018). Although ACL injuries are more common for female athletes than for male athletes (Montalvo et al., 2018), the injury mechanism is likely the same (Owusu-Akyaw et al., 2018). Therefore, a factor that is related to the injury mechanism in addition to being more common for female athletes has potential to be a true risk factor. Future research should examine a connection of the early peak VM waveform to kinematics observed during ACL injury, and conduct prospective cohort studies to establish a connection to injury risk.

### Strengths

This is to our knowledge the first study to have used cluster analysis techniques on 3D motion capture data. During motion capture, a number of different time series are calculated resulting in thousands of data points per measurement. Traditionally, these thousands have been reduced to a small number of single values such as local or global peaks (Leppanen et al., 2017; Torry et al., 2011) which can be input into a statistical model. Our results show that reducing different curve shapes of the knee VM to a single data point in such a manner results in many of the extracted data points being essentially unrelated to the timing of ACL injury. This likely weakens data analysis of such studies in terms of statistical power and the clinical relevance of the results.

### Limitations

Assessment of homogeneity of shape within each cluster was performed via visual inspection. This requires a certain level of clinical judgement which may not be reliable between different assessors. The reader is encouraged to examine the results of the clustering process using our data and analysis script from the online depository (see data availability statement).

The C-Index of our cluster analysis is reported, but currently there is no consensus on what constitutes a good C-Index or how the number of clusters should be decided. We used the first elbow of the C-Index graph, or a 0.05 cut-off of a smooth graph. The potential number of clusters in our data is 1025 unique shapes, some of which likely differ only in the exact location of local maxima or minima. It’s possible, but in our opinion unlikely, that using 6 initial clusters would yield the same 6 shapes presented as the basic shapes in the present study, or that using 100 initial clusters would yield superior results.

We have reported results from only 35 athletes in the adolescent cohort. The choice to use a subset of the available data was made due to the exploratory approach undertaken. A sample size of 35 is common in biomechanical studies, and since 20 trials are collected from each athlete the study is adequately powered for the chi-squared test. Future studies with larger cohorts are required to confirm the frequency of the early peak VM waveform.

## Conclusions and clinical relevance

This is to our knowledge the first study that demonstrates that clustering techniques are feasible to extract meaningful information from biomechanical data with relevance to a specific injury mechanism. A small number of distinct shapes of early stance phase knee VM curves exist and can be identified with a cluster analysis procedure. The early peak knee VM shape is consistent with the ACL injury mechanism, since the injury occurs early and the knee VM can strain the ACL. The sex-specific frequencies of the early peak shape in adolescence is consistent with the sex-specific difference in ACL injury incidence and may be a predisposing factor to injury. These findings should inform prospective risk factor studies as well as studies on kinematics related to the early peak waveform.

## Data Availability

A number of decisions have to be made by the authors during data processing and analysis. The field of cluster analysis has not reached consensus on how to select many important parameters during the cluster analysis process, including the number of clusters. In order to facilitate replication and auditing of the results presented, a data set that can be used to replicate the cluster analysis is available 10.5061/dryad.v8n3gv3. The data set includes the R code used to perform the cluster analysis, but no other information such as sex or age.

## Abbreviations

ACL: Anterior cruciate ligament
VM: Valgus moment
